# Fermented Dairy Products Modulate *Citrobacter rodentium*–Induced Colonic Hyperplasia

**DOI:** 10.1093/infdis/jiu205

**Published:** 2014-04-04

**Authors:** James W. Collins, Christian Chervaux, Benoit Raymond, Muriel Derrien, Rémi Brazeilles, Artemis Kosta, Isabelle Chambaud, Valerie F. Crepin, Gad Frankel

**Affiliations:** 1 MRC Centre for Molecular Bacteriology and Infection, Department of Life Sciences, Imperial College London, United Kingdom; 2 Danone Nutricia Research, Centre Daniel Carasso, Palaiseau; 3 C2R, Paris, France

**Keywords:** probiotic, *C. rodentium*, fermented dairy products, bioluminescence imaging, microbiota, DLIT-µCT

## Abstract

We evaluated the protective effects of fermented dairy products (FDPs) in an infection model, using the mouse pathogen *Citrobacter rodentium* (CR). Treatment of mice with FDP formulas A, B, and C or a control product did not affect CR colonization, organ specificity, or attaching and effacing lesion formation. Fermented dairy product A (FDP-A), but neither the supernatant from FDP-A nor β-irradiated (IR) FDP-A, caused a significant reduction in colonic crypt hyperplasia and CR-associated pathology. Profiling the gut microbiota revealed that IR-FDP-A promoted higher levels of phylotypes belonging to Alcaligenaceae and a decrease in Lachnospiraceae (*Ruminococcus*) during CR infection. Conversely, FDP-A prevented a decrease in *Ruminococcus* and increased Turicibacteraceae (*Turicibacter*). Importantly, loss of *Ruminococcus* and *Turicibacter* has been associated with susceptibility to dextran sodium sulfate–induced colitis*.* Our results demonstrate that viable bacteria in FDP-A reduced CR-induced colonic crypt hyperplasia and prevented the loss of key bacterial genera that may contribute to disease pathology.

Probiotics are increasingly being used as alternative therapies for inflammatory bowel diseases, such as ulcerative colitis and Crohn's disease, or as a treatment for antibiotic-resistant gastrointestinal infections [1–3]. Probiotic bacteria are typically consumed daily in the form of fermented dairy products (FDPs), supplements, or medical foods [1, 3], and are defined as live microorganisms that confer a health benefit to the host, aside from general nutrition [4]. Typically, probiotic bacteria are from the *Lactobacillus* and *Bifidobacterium* genera, and the majority of commercially available probiotic products contain multiple bacterial species in the form of supplements and FDPs. Members of the *Lactobacillus casei* group (*Lactobacillus paracasei* subspecies *paracasei* [*L. paracasei*] and *Lactobacillus rhamnosus* [*L. rhamnosus*]) are among the most widely marketed and characterized probiotic species. Few *Lactobacillus* species are likely to be permanent residents of the human gastrointestinal microbiota and are often transient colonizers found in fermented foods [5–8]. Therefore, dosing regimens and the activity of probiotic *Lactobacillus* strains during transit through the gastrointestinal tract is of paramount importance [6–8].

The innate ability of the intestinal microbiota to outcompete invading bacterial species, known as competitive exclusion or colonization resistance, is executed by a variety of mechanisms including the production of antimicrobial peptides [9] and compounds such as organic acids [10], enhancement of epithelial barrier function [11], antagonism of receptor sites present on the mucosal epithelium [12], competition for nutrients within the lumen of the gastrointestinal tract [13], inhibition of quorum sensing systems in pathogenic bacteria [14], and direct modulation of the host's immune system (including innate defenses such as mucin or defensin production) [15, 16]. However, the specific molecular mechanisms underlying these well-documented protective effects and the probiotic “effectors” that confer these effects are largely unknown.

*Citrobacter rodentium* (CR) is a mouse-specific pathogen that shares virulence factors and mechanism of colonization via attaching and effacing (A/E) lesions with the human pathogens enteropathogenic and enterohemorrhagic *Escherichia coli* [17, 18]. CR causes a self-limiting disease in C57Bl/6 mice, colonic epithelial cell hyperplasia, and transmissible colitis delineated by Th1 and Th17 immune responses [19]. The CR model has been widely adopted to study if probiotics can confer colonization resistance, or modulate colitis [15, 20–26]. These studies demonstrated that pretreatment of neonatal and adult mice with individual probiotic strains such as *Lactobacillus acidophilus*, *L. rhamnosus*, and *Lactobacillus helveticus* resuspended in phosphate-buffered saline (PBS) ameliorated CR-induced inflammation and colonic crypt hyperplasia [15, 16, 23], whereas Wu et al [26] showed that CR virulence was modulated if mice were pretreated with the probiotic yeast *Saccharomyces boulardii*. In this study, we evaluated the impact of formulations of FDPs on host–pathogen interactions and the intestinal microbiota in the context of CR infection.

## METHODS

### Bacterial Strains and Products

The bacterial strains (Table 1) used to make the FDPs at Danone Research were isolated from traditional dairy products and selected through in vitro screening of anti-infectious and immunomodulatory properties. Short and long fermentations were separated by at least 12 hours. The pH of each product was not buffered and was measured at the end of fermentation. The appearance, taste, and nutritional composition of the products and control were identical. FDPs were stored at 4°C. CR was grown as described [27]. Supernatant from FDP-A (S-FDP-A) was obtained by centrifugation (4000*g*, 4°C, 10 minutes) of 100 mL of the product and stored at −20°C. Fresh FDP-A was β-irradiated (IR-FDP-A) with a dosage of 20 kGy and stored at 4°C. Loss of viability was confirmed by plating.

**Table 1.** Composition of Fermented Dairy Products

Product	Product pH	Fermentation Time	Bacterial Strains
FDP-A	pH 3.8	Long	*L. paracasei* CNCM I-1518
			*L. paracasei* CNCM I-3689
			*L. rhamnosus* CNCM I-3690
			*L. delbrueckii* subsp *bulgaricus*
			*S. thermophilus*
FDP-B	pH 4.1	Short	*L. paracasei* CNCM I-1518
			*L. paracasei* CNCM I-3689
			*L. rhamnosus* CNCM I-3690
			*L. delbrueckii* subsp *bulgaricus*
			*S. thermophilus*
FDP-C	pH 3.8	Long	*L. paracasei* CNCMI-1518
			*L. delbrueckii* subsp *bulgaricus*
			*S. thermophilus*
Control product^a^	pH 3.8	NA	None

Abbreviations: FDP, fermented dairy product; NA, not applicable.

^a^ The control product was a sweetened nonfermented, acidified milk. The appearance, taste, and nutritional composition (proteins, carbohydrates, lipids, and energy) of the test products and control were identical.

### Treatment of Mice With FDPs and CR Infection

Pathogen-free female C57Bl/6 mice weighing 18–20 g were housed in HEPA-filtered cages with sterile bedding, food, and water. Experiments were repeated on 2 or 3 separate occasions with 6–8 mice per group. In separate experiments, mice were gavaged daily between 9 and 10 am without anesthesia with 200 µL of one of the FDPs, IR-FDP-A, S-FDP-A, control product [CP] or PBS for 10 days. At 2 pm on day 10 after initiation of FDP treatment, mice were infected by oral gavage with 200 µL of CR cultured overnight in Luria Bertani broth [20], and were given the FDPs for an additional 8 days. All animal experiments were performed in accordance with the Animals (Scientific Procedures) Act 1986 and were approved by the local ethical review committee.

### In Vivo Optical Imaging of *C. rodentium*–Infected Mice

Whole-animal bioluminescence imaging (BLI) was performed on days 4 and 8 postinfection (p.i.) using an IVIS 50 or IVIS Spectrum CT (PerkinElmer) [20, 28]. Regions of interest were identified and quantified, photons s^−1^ (total photon flux), using the appropriate version of Living Image software (PerkinElmer). Two representative mice from each group were imaged daily using diffuse light imaging tomography with integrated µCT imaging [20, 28].

### Sample Processing and Histological Analysis

Colonization was monitored by daily enumeration of viable bacteria per gram of feces [20]. At day 8 p.i., segments of terminal colon were removed and fixed in 10% buffered formalin [20]. Crypt hyperplasia was calculated by measuring the lengths of at least 20 well-oriented crypts from each section, from all of the mice. Additional colonic segments were fixed in 2.5% glutaraldehyde for transmission electron microscopy (TEM) and scanning electron microscopy (SEM). Histological damage scoring was determined as described elsewhere [26]. Five independent fields of view from one representative tissue section was graded from all of the mice and averaged to obtain a mean histological score. All histological sections were evaluated blindly.

### Indirect Immunofluorescence Staining and Electron Microcopy

Rabbit polyclonal antiserum was raised against β-irradiated *L. rhamnosus* CNCM I-3690 by Covalab. Indirect immunofluorescence was performed following heat-induced epitope retrieval, on formalin-fixed, paraffin-embedded sections. Chicken polyclonal anti-β*-*intimin and anti–*L. rhamnosus* antibodies were used to visualize CR and *L. rhamnosus*, respectively; DNA was counterstained with Hoescht 33342. Colonic tissues were processed for TEM and SEM as described previously [29].

### Fecal DNA Extraction

Forty-two fecal samples were collected from FDP-A– and IR-FDP-A–treated groups at the following time points: prior to FDP treatment (day –10), the day of CR infection (day 0), and 8 days after CR infection (day 8)*.* Fecal pellets were transferred to an RNA*later* solution (Ambion), homogenized, and volume-adjusted to a final fecal dilution of 1:10 (wt/vol). Two hundred microliters was added to 1 mL PBS and centrifuged for 5 minutes at 5000*g*. Supernatant was discarded and pellets stored at −80°C until extraction as described previously [30]*.* DNA was analyzed by quantitative polymerase chain reaction (qPCR) and 16S amplicon pyrosequencing.

### Quantification of Bacteria by qPCR

Primers and target loci are described in Supplementary Table 1. The Yakult Intestinal Flora-SCAN technology was used to perform qPCR as described previously [30, 31]. Reference strains (outlined in Supplementary Table 1) were used to establish standard curves and test primer specificity.

### Gut Microbiota 454 Analysis

V5 and V6 hypervariable 16S ribosomal RNA (rRNA) regions were amplified using primers 784F and 1061R (Supplementary Table 1) [32]. Sequencing was performed by DNAVision SA (Charleroi) on a 454 Life Sciences Genome Sequencer FLX instrument (Roche) using titanium chemistry and primer A.

### 16s rRNA Pyrosequencing Analysis

Analyses were performed using QIIME version 1.6 [33]. A total of 306 393 reads were obtained from 5 Multiplex 454 FLX regions and assigned to 42 samples after filtering according to the following quality criteria: size between 150 and 500 nt, quality >25 over a 50 base-pair window, no mismatch authorized in primers and barcode sequences, and absence of polymers >6 nt. A total of 225 096 reads were clustered into operational taxonomic units (OTUs) defined at 97% identity using cd-hit [34], and representative sequences for each OTU were aligned and taxonomically assigned using Greengenes version 11_04 database. ChimeraSlayer [35] was used to discard potential chimeric sequences, leading to a mean of 5039 ± 1213 (SD) reads per sample.

For α and β diversity, samples were rarefied to 3000 sequences per sample. α-Diversity (that measures diversity within samples) was assessed using rarefaction curves for richness (Chao1), and evenness (Shannon index) and numbers of observed OTUs (Supplementary Figure 2*A*–*C*); β-diversity (that measures diversity between samples) was performed on both weighted and unweighted Unifrac distances. Jackknife randomization for robustness evaluation was performed 10 times using 2700 randomly chosen sequences for each sample.

### Statistical Analyses

For the microbiota analysis, a selection of discriminant bacterial genera between different treatment groups was identified using an extension of a multivariate statistical analysis, sparse partial least squares discrimination analysis (sPLS-DA) [36]. To determine the difference between bacterial genera between 2 time points, the value of the last time point minus the value of the first time point for each population was used. Nonparametric Kruskal–Wallis test was then performed on the subspace of selected genera to confirm their differences between populations with a Benjamini–Hochberg multiple testing correction [37]. Wilcoxon Mann–Whitney tests without multiple testing correction were used to identify discriminant phyla and families and for qPCR analysis.

A one-way analysis of variance with group-specific variance and Tukey multiple-comparison posttest was used to analyze all other data, using commercially available software (GraphPad 5 and SAS 9.2); a *P* value of <.05 was taken to be significant.

## RESULTS

### The Effect of FDPs on Host–Pathogen Interactions

**Figure 1.**
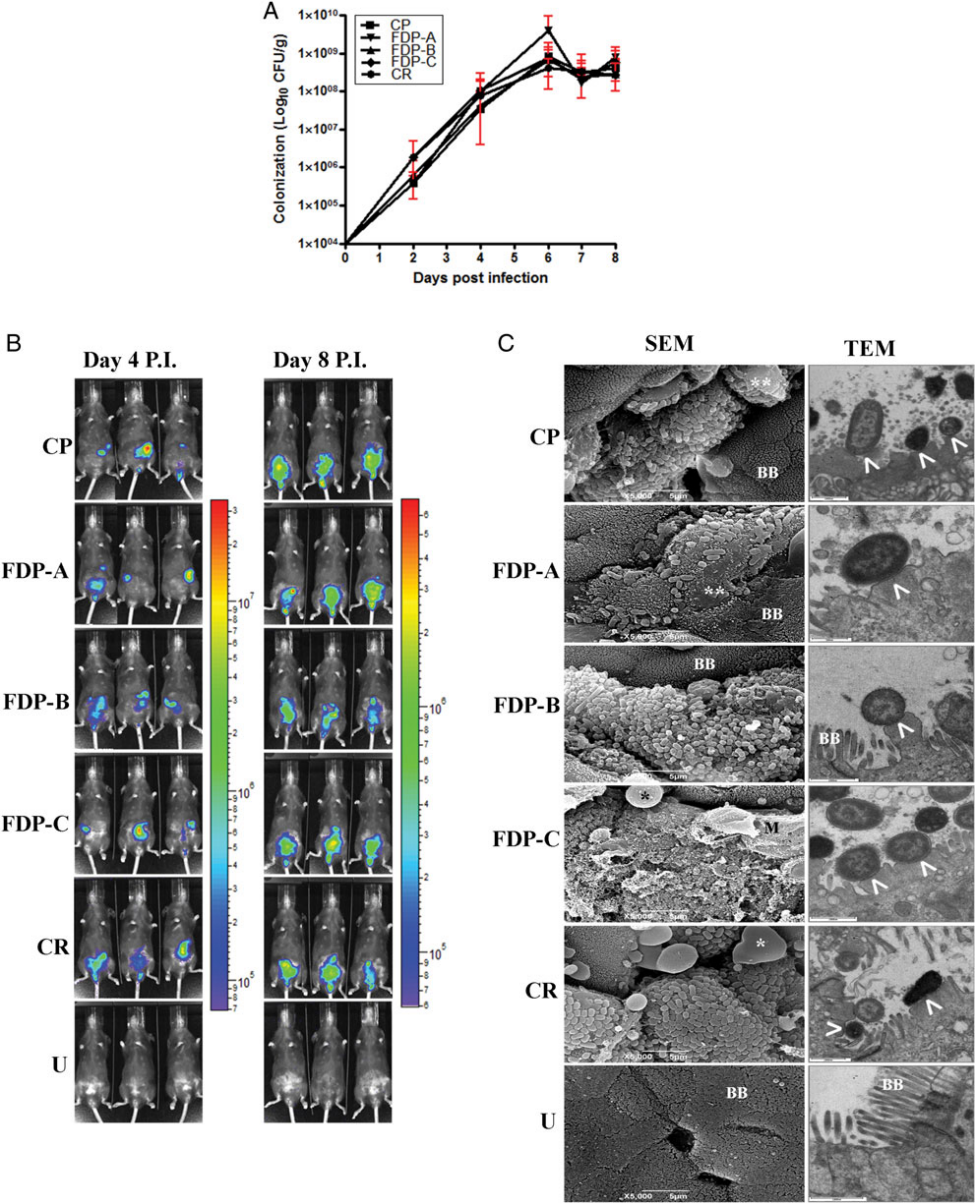
*A*, Quantification of *Citrobacter rodentium* (CR) colony-forming units (CFU) from stools over 8 days postinfection (p.i.) in the different treatment groups. *B*, In vivo optical imaging of a bioluminescent (BL) CR infection from 3 representative mice per treatment at days 4 and 8 p.i. *C*, Electron microscopy of terminal colon at day 8 p.i. reveals epithelial cell death (*) and attaching and effacing (A/E) lesions (arrowheads), irrespective of the fermented dairy product (FDP) treatment used. Abbreviations: BB, brush border; CFU, colony-forming units; CP, control product; SEM, scanning electron microscopy; TEM, transmission electron microscopy; U, untreated and uninfected.

We determined whether treatment with FDPs A–C could competitively exclude CR. None of the FDPs caused any significant reduction in CR colonization when evaluated by bacterial enumeration (Figure 1*A*) or BLI (Figure 1*B*), compared with the CP-treated group or the untreated control (CR). The spatial distribution of bioluminescent (BL) CR within infected mice was heterogeneous within a treatment group, due to the dynamic nature of the intestines within a live mouse (Figure 1*B*). The organ specificity of infection monitored by BLI was in line with previous reports [27], with the cecum heavily colonized at day 4 p.i. and cecum, colon, and rectum at day 8 p.i. (Figure 1*B*). None of the FDP treatments affected the organ specificity of infection (Figure 1*B*) or A/E lesion formation (Figure 1*C*).

**Figure 2.**
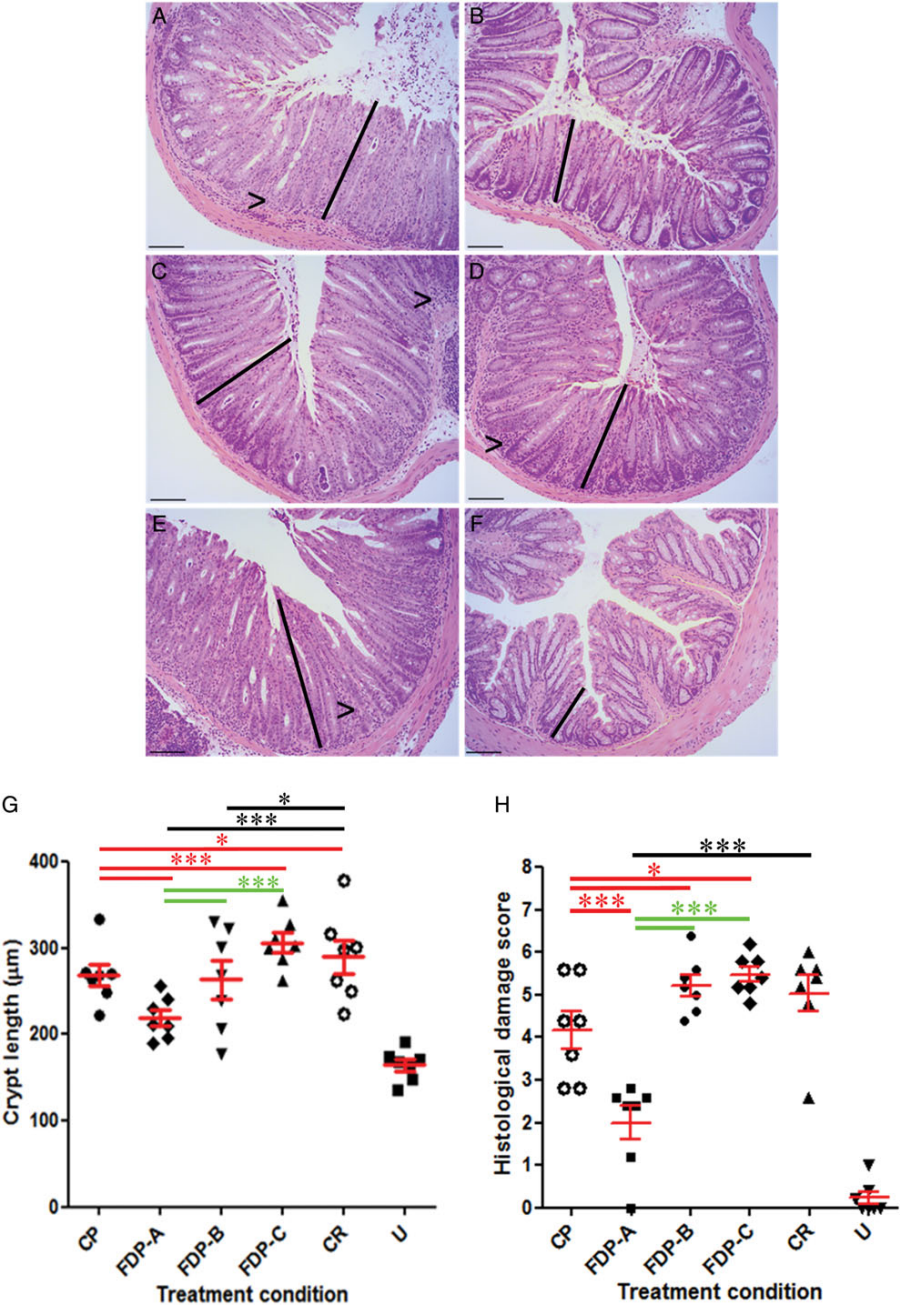
Histological analysis of *Citrobacter rodentium* (CR)–infected mice following treatment with (*A*) control product (CP), (*B*) fermented dairy product (FDP) A, (*C*) FDP-B, (*D*) FDP-C, (*E*) no treatment (*Citrobacter rodentium* [CR]), and (*F*) untreated and uninfected (U). FDP-A and FDP-B reduced lymphocyte accumulation in the lamina propria (arrowheads). Scale bar = 100 µm. *G*, Treatment of mice with FDP-A significantly reduced crypt hyperplasia compared with CP-treated mice (***P < .0001). *H*, Histological damage score demonstrating that FDP-A significantly reduced disease pathology compared with CR- and CP-treated groups (***P < .0001). FDP-A significantly reduced CR-associated pathology compared with FDP-B (***P < .0001). *P* values in red have been calculated compared with CP; *P* values in black have been compared to the CR group (*P < .05); *P* values in green are comparisons between FDPs.

Quantification of colonic crypt hyperplasia at day 8 p.i. demonstrated that CP (*P* < .0001) and FDP-B (*P* < .01) significantly reduced colonic crypt length (Figure 2*A*–*H*). However, treatment with FDP-A was significantly more effective than CP and FDP-B (Figure 2*G* and 2*H*) and was therefore selected for further study.

### Viable Bacteria in FDP-A Reduce CR-Associated Pathology

**Figure 3.**
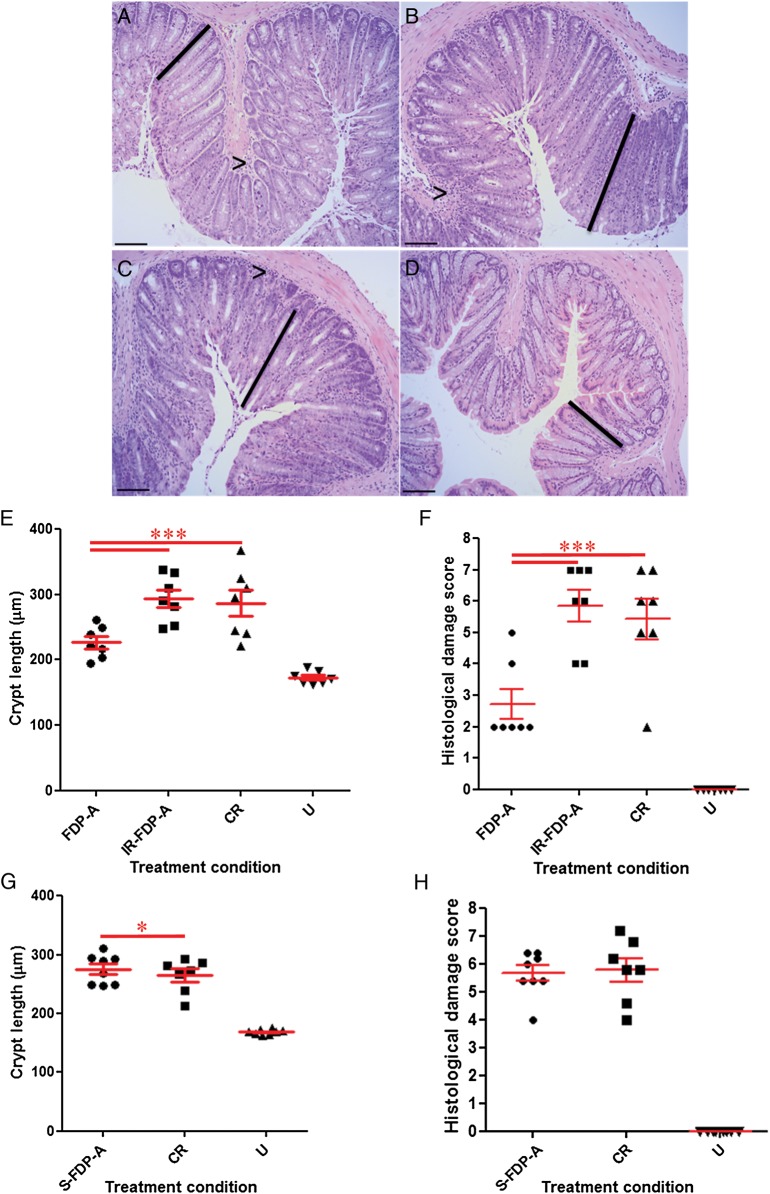
Quantification of crypt hyperplasia following treatment of mice with (*A*) fermented dairy product (FDP) A or (*B*) β-irradiated (IR) FDP-A, (*C*) no treatment (*Citrobacter rodentium* [CR]), and (*D*) untreated uninfected (U). *E,* FDP-A significantly reduced crypt hyperplasia compared with IR-FDP-A (***P < .0001) and qualitatively reduced lymphocyte accumulation in the lamina propria (arrowheads). IR-FDP-A treatment did not significantly reduce crypt hyperplasia compared with CR-infected mice (*P* > .05). *F*, Histological damage score demonstrating that FDP-A, but not IR-FDP-A, significantly reduced CR-associated pathology (***P < .0001). *G*, Treatment with supernatant from S-FDP-A significantly increased crypt hyperplasia compared with CR-treated or untreated and uninfected (U) mice (*P < .05). *H*, Histological damage score demonstrated that S-FDP-A treatment did not significantly alter CR associated pathology compared with CR-treated mice (*P* > .05). Scale bar = 100 µm.

To determine the functional component of FDP-A responsible for causing a reduction in colonic crypt hyperplasia, mice were pretreated for 10 days with FDP-A, IR-FDP-A, or S-FDP-A; infected with CR; and administered the products daily for the duration of the study. Neither treatment caused a reduction in colonization or organ specificity of infection (Supplementary Figure 1). Quantification of colonic crypt hyperplasia demonstrated that FDP-A but not IR-FDP-A or S-FDP-A significantly reduced crypt length (*P* < .0001; Figure 3*A*–*H*). Moreover, the pathology score of mice treated with IR-FDP-A or S-FDP-A was not significantly reduced (*P* > .05; Figure 3*A* and 3*C*), demonstrating that health benefits require viable bacteria in the FDP.

### *Lactobacillus rhamnosus* Present in FDP-A Is Associated With Colonic Epithelial Cells

**Figure 4.**
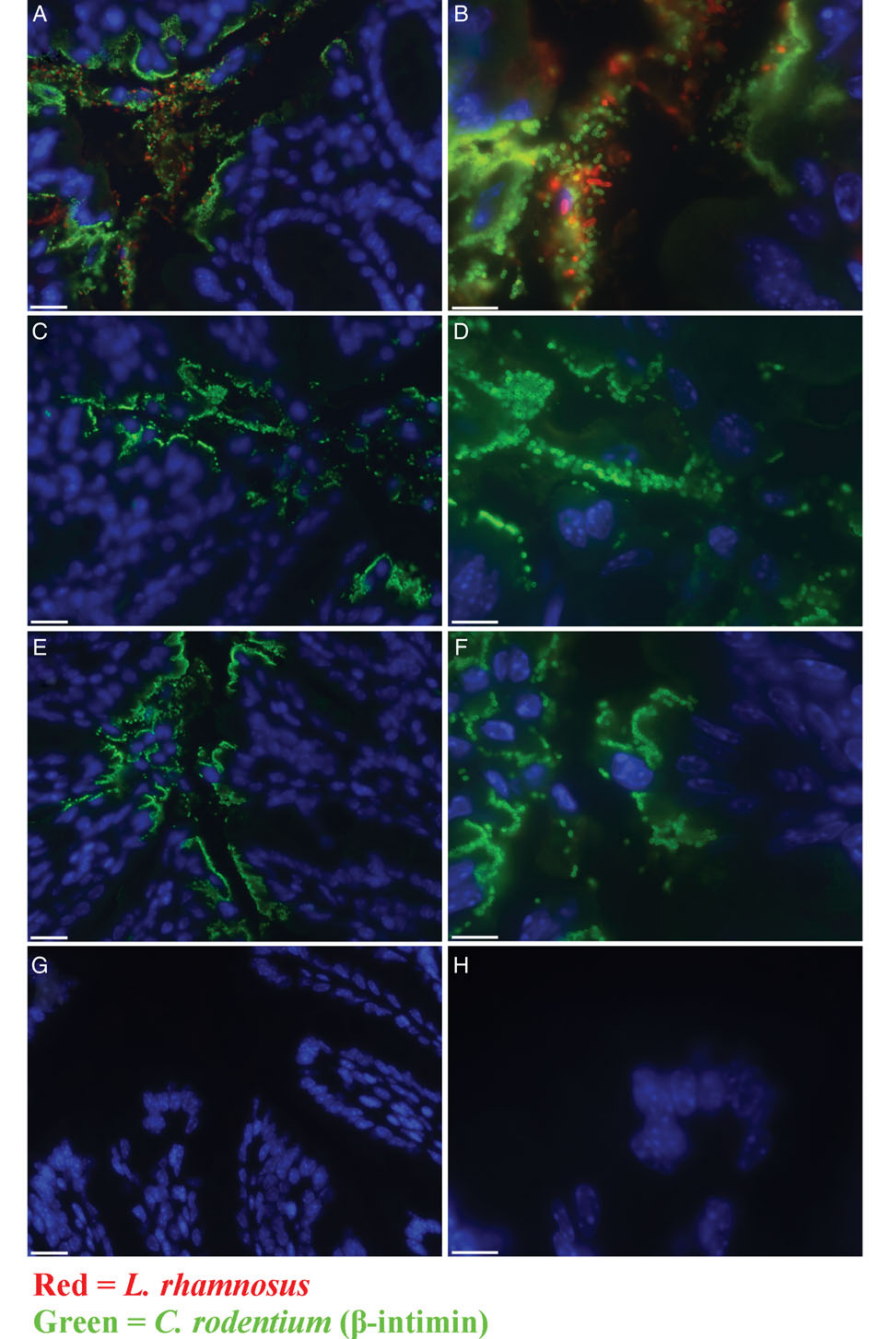
Indirect immunofluorescence using anti β-intimin (green) and anti–*Lactobacillus rhamnosus* (red). *A* and *B*, Fermented dairy product (FDP)–A treated and *Citrobacter rodentium* (CR) infected. (*C* and *D*), Irradiated FDP-A treated and CR infected. (*E* and *F*), No treatment and CR-infected and (*G* and *H*) untreated and uninfected (U). CR and *Lactobacillus rhamnosus* were observed associated to the epithelial layer lining the lumen of the colon. *Lactobacillus rhamnosus* was only found in the intestinal lumen, or associated with colonic epithelial cells following FDP-A treatment. The distribution of CR associated with the colonic mucosa was not affected by FDP treatment. Scale bars = 20 µm (left column) and 10 µm (right column).

We visualized the distribution of *L. rhamnosus* on the colonic mucosa following treatment with FDP-A or IR-FDP-A and CR infection (day 8 p.i.). *L. rhamnosus* could only be found in FDP-A–treated mice, but not in IR-FDP-A–treated mice (Figure 4*A* and 4*B*). Qualitative assessment of *L. rhamnosus* distribution showed multiple bacteria associated with colonic epithelial cells lining the lumen and colocalization with CR on infected epithelial cells (Figure 4*A* and 4*B*). Small numbers of *L. rhamnosus* could be visualized at the bottom of the crypts and within goblet cells (data not shown). *L. rhamnosus* was also present in high numbers within the intestinal lumen (Figure 4*A* and 4*B*). Evaluating whether the treatment altered CR distribution within the colonic mucosa revealed that neither FDP-A nor IR-FDP-A affected the pattern of CR colonization of epithelial cells lining the colonic lumen and crypts (Figure 4*A*–*G*).

### Quantification of FDP-A Bacteria in Feces and Modulation of the Intestinal Microbiota

**Figure 5.**
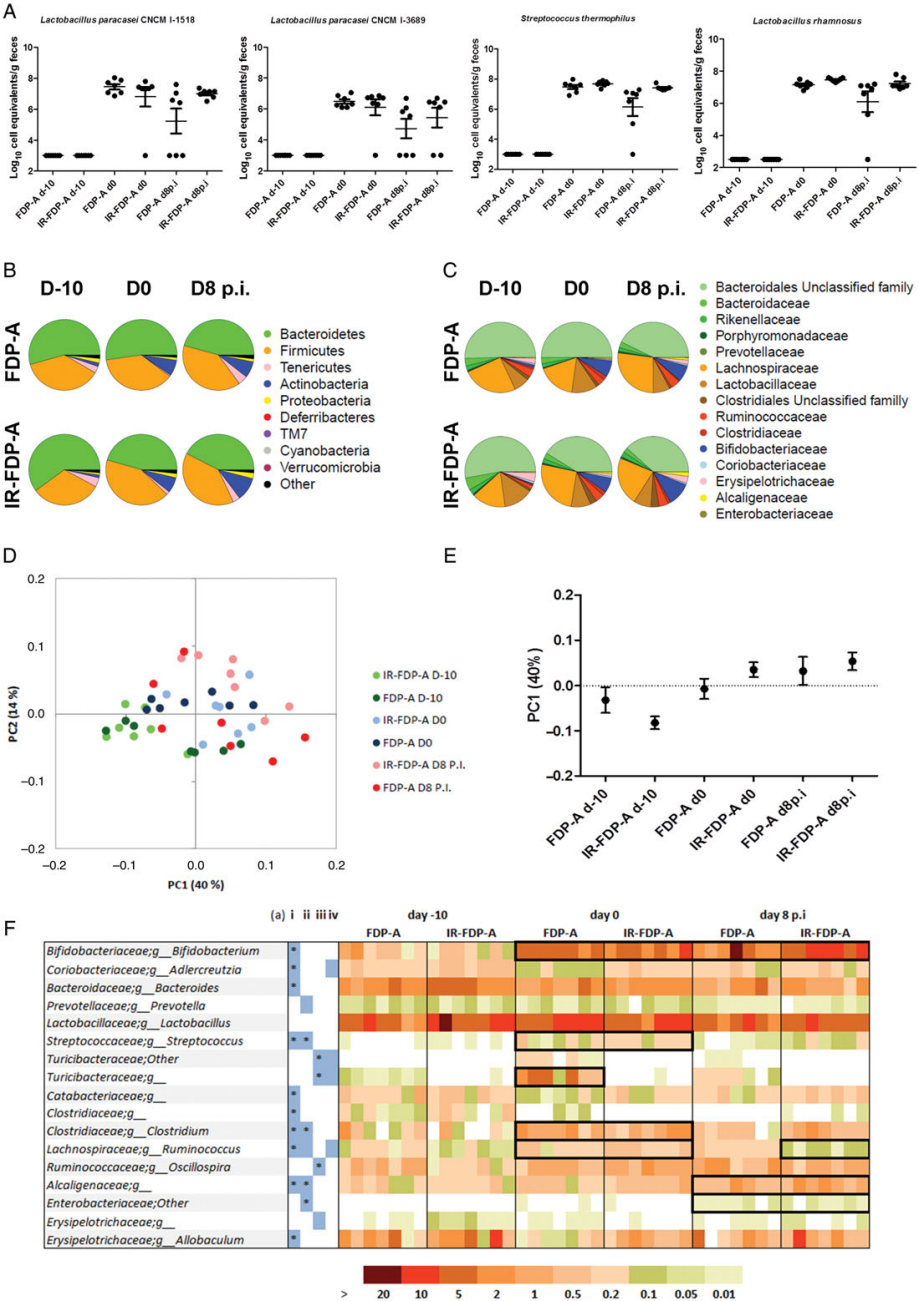
Microbiota profiling of stool samples by 16S rRNA gene pyrosequencing and quantitative polymerase chain reaction (qPCR). Samples were taken before treatment (day –10 [d-10]), before infection (day 0 [d0]), and 8 days postinfection (d8 p.i.). *A*, Evaluation of colonization of 4 strains present within fermented dairy product (FDP) A and β-irradiated (IR) FDP-A by qPCR. Taxa abundance profiles at (*B*) the phylum level and (*C*) the family level. *D* and *E*, Representation of 2D and 1D principal coordinates analysis of weighted Unifrac distances. *F*, Discriminant genera identified by sparse partial least squares multivariate analyses. Blue-colored boxes indicate discriminant genera in the corresponding comparisons (i) between day –10 and day 0 for both products, (ii) between day 0 and day 8 p.i. for both products, (iii) between FDPs for change after consumption (day 0 – day –10), or (iv) after infection (day 8 p.i. – day 0). Bold black frames focus on results described in the text, and stars in the blue boxes indicates significant population differences identified using a nonparametric Kruskal–Wallis test. “Other” indicates sequences that could not be attributed to a lower taxonomic level; *g_* indicates sequences assigned to bacteria unclassified at the genus level.

We quantified the levels of 4 bacterial strains present in FDP-A by qPCR prior to treatment with the FDPs (day –10), 10 days after treatment with FDP-A or IR-FDP-A (day 0), and 8 days following CR infection (day 8 p.i.). This was done at either the strain level (*L. paracasei* strains CNCM I-1518 and CNCM I-3689) or species level (*L. rhamnosus* and *Streptococcus thermophilus* [*S. thermophilus*]) (Figure 5*A*). None of the bacterial species found in FDP-A were detected in feces before administration of the product. We detected similar levels of the bacterial species in the FDP-A– and IR-FDP-A–treated groups on days 0 and 8 after CR infection, suggesting that DNA from dead bacteria in the IR-FDP-A group was detected, with levels of different strains ranging from 5 × 10^6^ to 1 × 10^8^ cell equivalents per gram of feces.

**Figure 6.**
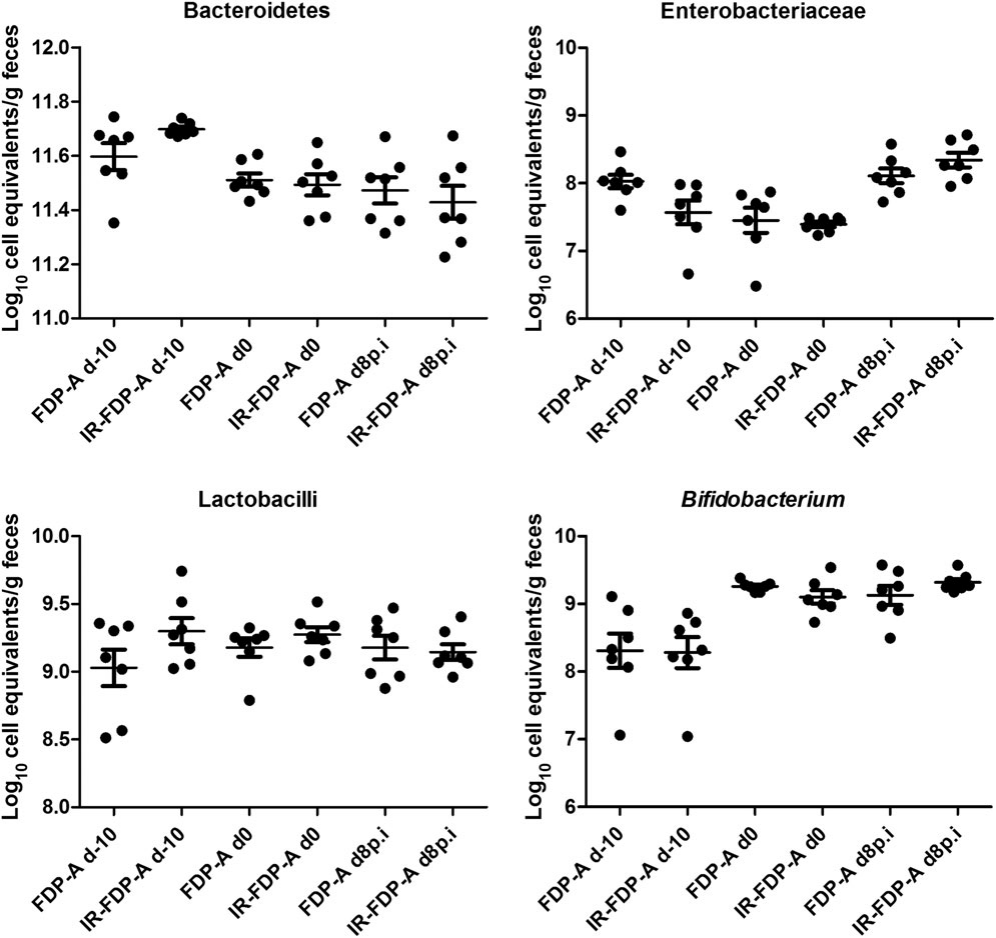
Taxa abundance profiles according to treatment groups and time points in cell equivalents per gram of feces were calculated using quantitative polymerase chain reaction. Abbreviations: FDP, fermented dairy product; IR, β-irradiated; p.i. postinfection.

We hypothesized that viable bacteria present in FDP-A may alter the intestinal microbiota and that these changes could be responsible for the observed reduction in CR-induced colonic hyperplasia and pathology. To test this, fresh feces were collected at the different time points, and the microbial community present was profiled by 16S rRNA gene pyrosequencing (Figure 5*B*–*F*) and qPCR (Figures 5*A* and 6). The 16S rRNA gene sequences were clustered into OTUs (97% identity), and representative sequences were aligned and used to calculate Unifrac distances between each sample pairs. Principal coordinates analysis of the weighted Unifrac distances (Figure 6*D*) indicated that samples from day –10 (baseline) clustered together and were more similar than samples from later time points (day 0 and day 8 p.i.). However, neither IR-FDP-A nor FDP-A were identified as discriminating factors following principal coordinates analysis, despite their good representation of the total variability (54%; Figure 5*E*). Therefore, the major modification of the intestinal microbiota in this study occurred between day –10 and day 0 (the administration phase).

Prior to FDP treatment (day –10), the microbiota profiles from the phylum level of the 2 treatment groups were similar, apart from Proteobacteria representing 2% (±1%) in the FDP-A group and 1% (±0.28%) in the IR-FDP-A group (Figure 5*B*). Treatment of mice for 10 days with FDP-A or IR-FDP-A caused a significant increase in the proportion of Actinobacteria in both treatment groups, from a mean of 1.65% (±1.35%) at day –10 to 7.28% (±1.63%) at day 0 in the FDP-A group and from 1.29% (±0.37%) at day –10 to 7.1% (±3.69%) at day 0 in the IR-FDP-A group. At a lower taxonomic level, there was an increase at day 0 in the Bifidobacteriaceae family detected by 16S rRNA sequencing for both groups (*P* = .002) and in the *Bifidobacterium* genus detected by qPCR (*P* = .002 and *P* = .004 for FDP-A and IR-FDP-A, respectively) (Figures 5*B*, 5*C*, and 5*F* and 6). The increase in *Bifidobacterium* species persisted at day 8 p.i. in both treatment groups. In addition, both sequencing analysis and qPCR indicated a significant (*P* = .0049 and *P* = .0022) decrease in the relative abundance of Bacteroidetes following intake of IR-FDP-A (Figures 5*B* and 6), whereas Proteobacteria and Actinobacteria were higher in IR-FDP-A at day 8 p.i.

At the family level, Bifidobacteriaceae and Clostridiaceae increased following the consumption period, with an increase in Alcaligenaceae and Enterobacteriaceae observed in both groups during infection. However, Alcaligenaceae reached significantly (*P* = .04) higher levels at day 8 p.i. in IR-FDP-A (1.9% [±0.93%] vs 1% [±0.73%] in FDP-A; Figure 5*C* and 5*F*).

### FDPs Induce Discriminant Changes at Genus Level During CR Infection

Multivariate analysis (sPLS-DA) was performed to investigate global microbiota changes between day –10 and day 0 (Supplementary Figure 2*D* and 2*F* and Figure 5*F*) and then between day 0 and day 8 p.i. (Supplementary Figure 2*E* and 2*G* and Figure 5*F*) to identify bacterial phylotypes moving either concomitantly or specifically in both FDP-treated groups. All analysis achieved at least 85% accuracy in discriminating 2 different groups.

#### Consumption of FDPs

During the administration period (day –10 to day 0), for both FDPs, *Bifidobacterium*, *Clostridium*, unclassified genera from Alcaligenaceae, *Streptococcus*, and *Ruminococcus* were increased, whereas *Bacteroides*, *Allobaculum*, unclassified Clostridiaceae, and *Adlercreutzia* decreased (Figure 5*F*, i). An unclassified Turicibacteraceae was the only phylotype that discriminated between FDP-A and IR-FDP-A during this period. This family was almost absent from the IR-FDP group, but was detected in FDP-A–treated mice at very low levels at day –10 (0.07% corresponding to 1 or 2 reads).

#### CR Infection

In both treatment groups, we observed an increase in unclassified Alcaligenaceae (Figure 5*F*, ii) and a decrease in *Streptococcus* and *Clostridium.* Importantly, *Ruminococcus* (Lachnospiraceae family) and, to a lesser extent, *Adlercreutzia*, were discriminant between the 2 treatment groups between day 0 and day 8 p.i. (Figure 5*F* and Supplementary Figure 2*G*), and these 2 taxa decreased in IR-FDP-A at day 8 p.i. and were either stable or slightly increased in FDP-A at the same time point.

## DISCUSSION

This study is the first to evaluate whether a range of FDPs could affect the outcome of infectious colitis using a dosing regimen that mimics typical human consumption, in a murine infection model. In an initial screen of FDPs, our results demonstrated that daily prophylactic administration of FDP-A, FDP-B, and FDP-C did not prevent bacterial colonization or A/E lesion formation, or alter tissue specificity within the gastrointestinal tract. Studies using prophylactic administration of single strains of probiotic bacteria, or yeast resuspended in PBS, prior to CR infection significantly reduced the total bacterial load during infection [16, 22, 23, 26, 38]. These differences could be due to the different formulations, strain compositions, dosing regimens, and mouse strains employed.

In addition, probiotic bacteria have been demonstrated to alter the intestinal inflammatory response following chemical- or CR-induced colitis [15, 16, 23–26]. Pretreatment of mice with FDP-A and -B significantly reduced colonic hyperplasia, which is routinely used as a marker of intestinal inflammation in the CR model. Importantly, *L. rhamnosus* GG has been demonstrated to reduce cell cycle progression in cancer cells in vitro and, following a single treatment, induces epithelial cell proliferation in *Drosophila melanogaster*, mice, and humans [39–41]. Collectively, these data suggest that treatment of mice with *Lactobacillus* species (including *L. rhamnosus*) in the form of FDP-A can antagonize colonic epithelial cell hyperproliferation.

Secreted proteins p40 and p75 from *L. rhamnosus* GG have been shown to modify dextran sodium sulphate– or oxazolone-induced colitis and reduce colonic hyperplasia [42]. In this study, prophylactic treatment with FDP-A and -B demonstrated varying efficacy in the reduction of colonic hyperplasia, with FDP-A being the most effective. FDP-A and FDP-B contain exactly the same bacterial cultures and differ only in their fermentation time and culture pH, suggesting that the phenotype might involve either secreted metabolites or the physiology of the strains. However, as the supernatant from FDP-A did not reduce colonic hyperplasia or CR-associated pathology, the beneficial component of FDP-A is unlikely a secreted compound, unless it has a short half-life. Therefore, the fact that longer fermentation is needed to obtain greater protection suggests that protection is conferred by metabolic or proteomic/glycomic changes to the surface of bacteria in the product.

Quantification of bacteria present in the products in fecal samples demonstrated that *Lactobacillus* species and *S. thermophilus* were found in mice treated with either active or irradiated products following CR infection, indicating that DNA was still detected in the feces when mice were fed with dead bacteria. In contrast with our findings, it was reported that *Lactobacillus* species were decreased during CR infection [43]. However, it is likely that the daily administration of lactobacilli in FDPs masked the loss of endogenous *Lactobacillus* species. Importantly, we found that bacteria present in FDP-A, but not IR-FDP-A, were associated with CR-infected epithelial cells, which might be important for the protective effect of FDP-A.

Currently, there is much conjecture regarding what constitutes a “healthy” microbiota; however, it is well established that the loss of key bacterial genera can modify intestinal homeostasis and alter the immune response to gastrointestinal infections [44]. Profiling changes to the composition of the intestinal microbiota following FDP treatment demonstrated a large increase of *Bifidobacterium* species for both FDP-A– and IR-FDP-A–treated mice, suggesting that the composition of FDP-A, and not viable bacteria, is responsible; a similar phenotype was described in a humanized rat model fed *L. paracasei*–fermented milk [45]. We hypothesized that the reduced colonic hyperplasia in FDP-A–treated mice was associated with changes to key bacterial taxa that modulate mucosal homeostasis. Consumption of IR-FDP-A promoted higher levels of phylotypes belonging to Alcaligenaceae and a decrease in Lachnospiraceae (*Ruminococcus*) during CR infection, which was prevented in the FDP-A–treated group. Consumption of FDP-A induced a strong increase in Turicibacteraceae (*Turicibacter*), which decreased during CR infection but was below the detection limit in IR-FDP-A–treated mice. Importantly, loss of *Ruminococcus* and *Turicibacter* has been associated with susceptibility to dextran sodium sulphate–induced colitis [46]. In addition, we observed an increase in the Alcaligenaceae family in IR-FDP-A–treated mice, which has been associated with immunomodulation in mice [47]. In future studies, we intend to use germ-free mice, or mice with a reduced complexity microbiota, to investigate how genera identified through microbial profiling contribute to colonic hyperplasia during CR infection and FDP treatment.

Collectively, these data demonstrate that FDP-A modifies murine intestinal homeostasis following CR infection through an active process, which requires live bacteria and is independent of stable secreted bacterial molecule(s), highlighting the importance of consuming viable fermented products. Moreover, the reduction in colonic hyperplasia is not caused by FDP-C, and therefore the protective effects are unique to the combination of strains within FDP-A, demonstrating the importance of using fermented products containing multiple probiotic species.

## Supplementary Data

Supplementary materials are available at *The Journal of Infectious Diseases* online (http://jid.oxfordjournals.org/). Supplementary materials consist of data provided by the author that are published to benefit the reader. The posted materials are not copyedited. The contents of all supplementary data are the sole responsibility of the authors. Questions or messages regarding errors should be addressed to the author.

Supplementary Data
