# Supplementary material for: Fermented Dairy Products Modulate Citrobacter rodentium–Induced Colonic Hyperplasia
Source: J Infect Dis. 2014 Apr 4;210(7):1029–41. doi: 10.1093/infdis/jiu205 (PMC4157696; doi:10.1093/infdis/jiu205)
Supplement: Supplementary Data [file supp_210_7_1029__index.html]

Fermented Dairy Products Modulate Citrobacter rodentium–Induced Colonic Hyperplasia — Fermented Dairy Products Modulate Citrobacter rodentium–Induced Colonic Hyperplasia — Supplementary Data 

# Fermented Dairy Products Modulate *Citrobacter rodentium*–Induced Colonic Hyperplasia

## Supplementary Data

Supplementary Data

**Files in this Data Supplement:**

- Supplementary Data - Docx file
- Supplementary Table 1 - docx file
