# Supplementary material for: Fermented Dairy Products Modulate Citrobacter rodentium–Induced Colonic Hyperplasia
Source: J Infect Dis. 2014 Apr 4;210(7):1029–41. doi: 10.1093/infdis/jiu205 (PMC4157696; doi:10.1093/infdis/jiu205)

**Fig S1.** **a.** Quantification of CR colony forming units from stools at day 7 p.i. **b.** Quantification of BL from the whole animal (abdominal region) in photons/second/centimeter squared/steradian (p/s/cm^2^/sr) at day 8 p.i. **c.** and **d.** *Ex vivo* quantification of BL (p/s) from colonic and caecal tissues at day 8 p.i., respectively. **e**. *In vivo* imaging of BL CR from 3 representative mice per treatment at day 8 p.i., **f.** DLIT-µCT scan of a of BL CR from one representative mouse per group monitored at days 3 and 8 p.i. Red arrow indicates caecal colonization; the blue arrowhead indicates colonic colonization. **g.** *Ex vivo* imaging of the murine gastrointestinal tract at day 8 p.i., **h.** *Ex vivo* imaging of a caecum from an IR-FDP-A treated mouse with the luminal contents removed and mucosal surface exposed. The black arrowhead delineates heavy CR colonization of the caecal patch.

**Fig S2**. Evaluation of Alpha diversity at each time point. Dot plots representing rarefication curves for **a**. the number of observed OTUs, **b**. Chao1 (Richness) and **c**. Shannon (evenness) diversity indexes. **d-g**. Representation of multivariate analyses of microbiota composition from the 42 samples using sPLS. The left panel represents a 2 dimension projection of the samples. The right panel represents the contribution of each of the original variables. Variables localized between the two correlation circles contribute to sample discrimination. **d**. before (green dots) and after (blue dots) FDP consumption. **e.** before (d0, blue dots) and after (d8 p.i, red dots) CR infection. **f**. and **g**. FDP-A (dark symbols) and IR-FDP-A (light symbols) groups for the changes measured between d-10 and d0 (panels f, blue dots) and d0 and d8 p.i (panel g, red dots).

**Table S1.** Primers and reference strains.


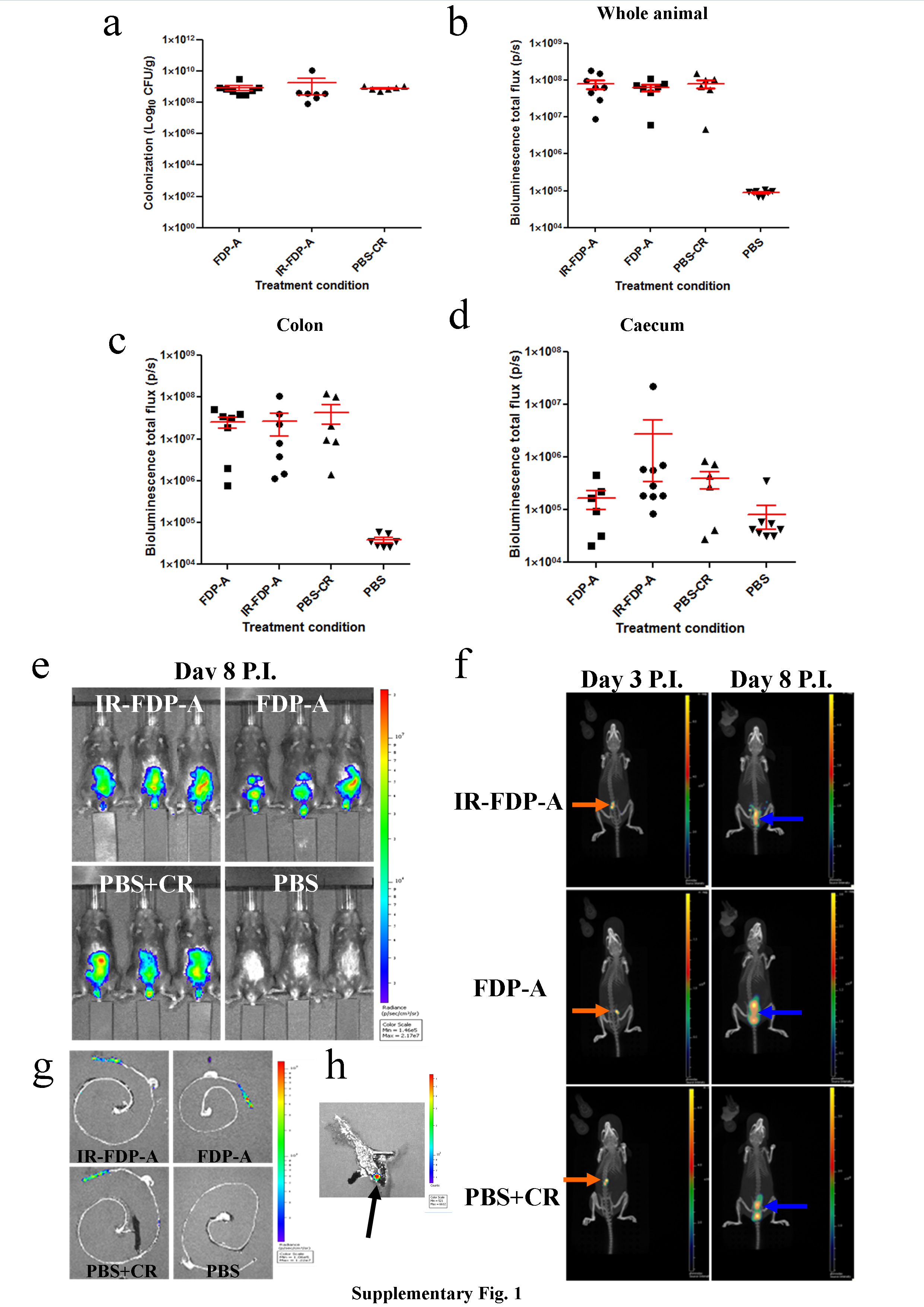

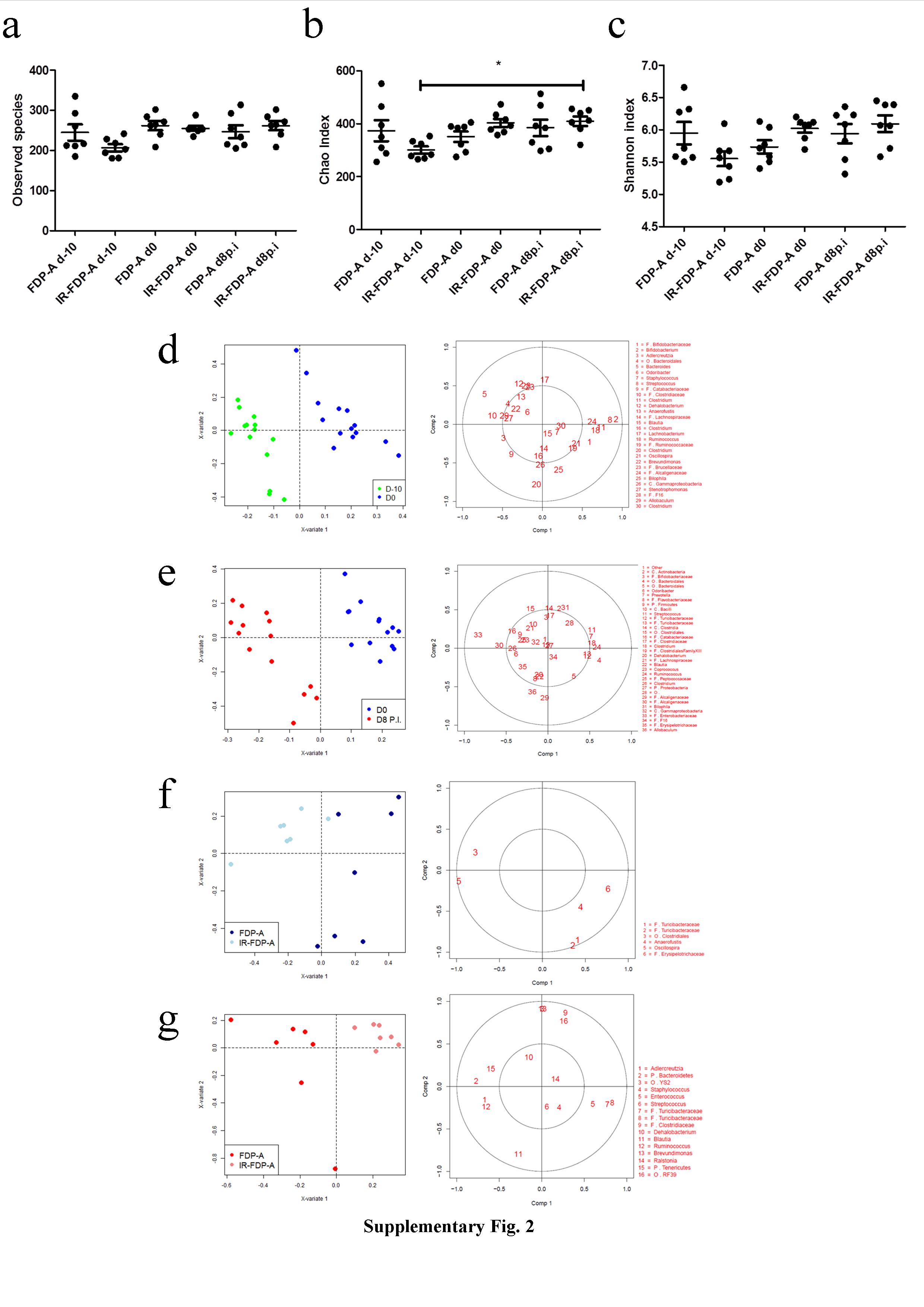

Supplement: Supplementary Data [file supp_jiu205_jiu205supp_data.docx]
