# Supplementary material for: Fermented Dairy Products Modulate Citrobacter rodentium–Induced Colonic Hyperplasia
Source: J Infect Dis. 2014 Apr 4;210(7):1029–41. doi: 10.1093/infdis/jiu205 (PMC4157696; doi:10.1093/infdis/jiu205)
Supplement: Supplementary Data [file supp_jiu205_jiu205supp_table.docx]

**Supplementary Table S1**

| **Target** | **Target locus** | **Primer**  **name** | **Primer sequence (5'-3')** | **454 adaptor sequence** | **Reference strain used** | **Annealing temp (°C)** | **Product size (bp)** | **Genetic material used** | **Ref or source** |
| --- | --- | --- | --- | --- | --- | --- | --- | --- | --- |
| Bacteria | V5 and V6 16S rRNA | 784F | AGGATTAGATACCCTGGTA | Titanium adaptor A and Barcode sequence CCATCTCATCCCTGCGTGTCTCCGACTCAG | NA | 56 | 270 | DNA | Andersson *et al*., 2008 |
|  |  | 1061R | CRRCACGAGCTGACGAC | Titanium adaptor B and barcode sequence CCTATCCCCTGTGTGCCTTGGCAGTCTCAG | NA |  |  |  |  |
| Bacteria | 16S rRNA | Uni331F | TCCTACGGGAGGCAGCAGT |  | *Bifidobacterium longum* ATCC 15707T | 60 | 466 | DNA | Nadkarni *et al*., 2002 |
|  |  | Uni797R | GGACTACCAGGGTATCTAATCCTGTT |  |  |  |  |  |  |
| *Lactobacillus* | 16S rRNA | Lacto 05 | AGCAGTAGGGAATCTTCCA |  | *Lactobacillus acidophilus* ATCC 4356T | 60 | 375 | DNA | Sokol *et al*. 2008 |
|  |  | Lacto 04 | CGCCACTGGTGTTCYTCCATATA |  |  |  |  |  |  |
| *Bifidobacterium* | 16S rRNA | g-Bifid153-F | CTCCTGGAAACGGGTGG |  | *Bifidobacterium longum* ATCC 15707T | 55 | 552 | DNA | Matsuki *et al.*, 2004 |
|  |  | g-Bifid699-R | GGTGTTCTTCCCGATATCTACA |  |  |  |  |  |  |
| Enterobacteriaceae | 16S rRNA | En-Isu3F | TGCCGTAACTTCGGGAGAAGGCA |  | *Escherichia coli* ATCC 11775T / DSM 30083T | 60 | 428 | DNA | Matsuki *et al*., 2007 |
|  |  | En-Isu3'R | TCAAGGCTCAATGTTCAGTGTC |  |  |  |  |  |  |
| Bacteroidetes | 16S rRNA | AllBac-F | GAGAGGAAGGTCCCCCAC |  | *Bacteroides thetaiotaomicron* ATCC 29148 / DSM 2079T | 60 | 106 | DNA | Layton *et al*, 2006 |
|  |  | AllBac-R | CGCTACTTGGCTGGTTCAG |  |  |  |  |  |  |
| *Lactobacillus rhamnosus* | 16S rRNA | Lrham01 | AAGTCGAACGAGTTCTGATTATTGAAA |  | *Lactobacillus rhamnosus* CNCM I-3690 | 60 | 119 | DNA | Firmenesse *et al*.2008 |
|  |  | Lrham02 | TCCAAATGTTATCCCCCACTTAAG |  |  |  |  |  |  |
| *Lactobacillus paracasei* CNCM I-1518 | CRISPR | OFF 2201 | GTTAGCACCGCTTAAAGACG |  | *Lactobacillus paracasei* CNCM I-1518 | 60 | 237 | DNA | this study |
|  |  | OFF 2202 | GCCATAAGCGTGTTAGCCG |  |  |  |  |  |  |
| *Lactobacillus paracasei* CNCM I-3689 | cas gene | OFF 3040 | TTATCGATCCCAAGCTGGAC |  | *Lactobacillus paracasei* CNCM I-3689 | 60 | 112 | DNA | this study |
|  |  | OFF 3041 | ATCAGCGGTCCTTCAACATC |  |  |  |  |  |  |
| *Streptococcus thermophilus* | 16S rRNA | St1 | TTATTTGAAAGGGGCAATTGCT |  | *Streptococcus thermophilus* CNCM I-1630 | 55 | 281 | DNA | Furet *et al*. 2003 |
|  |  | St2 | GTGAACTTTCCACTCTCACAC |  |  |  |  |  |  |
